# Supplementary material for: Magnetic resonance colonography assessment of acute trinitrobenzene sulfonic acid colitis in pre-pubertal rats
Source: PLoS One. 2021 Nov 4;16(11):e0259135. doi: 10.1371/journal.pone.0259135 (PMC8568272; doi:10.1371/journal.pone.0259135)
Supplement: Attachment — Submitted filename: Response to reviewers_RMLCD.docx [file pone.0259135.s001.docx]

Claire Dupont-Lucas

Department of Paediatrics

Gastroenterology, hepatology and nutrition

Caen University hospital

Avenue Côte de Nacre

CS 30001

14033 Caen cedex 9

France

Email: dupont-c@chu-caen.fr

Rosanna Di Paola

Academic Editor

PLOS ONE

Object: Response to reviewers

Caen, August 28^th^, 2021

Dear Dr Di Paola, Dear Reviewer,

We thank you for taking into consideration our article entitled “Magnetic Resonance Colonography assessment of Acute TNBS Colitis in pre-pubertal rats” and thank you for your comments.

Please find attached the revised manuscript, with and without tracked changes.

We also uploaded the Figures after checking them through the PACE system.

Here are the point-by-point replies to the Journal Requirements:

1. Please ensure that your manuscript meets PLOS ONE's style requirements, including those for file naming. The PLOS ONE style templates can be found at 
<https://journals.plos.org/plosone/s/file?id=wjVg/PLOSOne_formatting_sample_main_body.pdf> and 
<https://journals.plos.org/plosone/s/file?id=ba62/PLOSOne_formatting_sample_title_authors_affiliations.pdf>

=> We have verified that the manuscript met PLOS ONE’s style requirements. In particular we added indents for the first line of each paragraph (pages 2-17) and added the current address of one author (page 1).

2. We note that the grant information you provided in the ‘Funding Information’ and ‘Financial Disclosure’ sections do not match.

When you resubmit, please ensure that you provide the correct grant numbers for the awards you received for your study in the ‘Funding Information’ section.

=> we have corrected the name of the funder in the Funding information section

Also, we would like to amend the Role of funder statement as follows:

« This work was supported by the European Union and Normandy Regional Council. Europe contributes to Normandy through the European Regional Development Fund (ERDF). One of the authors (MP) received a grant from Nestlé Health Science given by SFNCM (Société Francophone Nutrition Clinique et Métabolisme).
The funders had no role in study design, data collection and analysis, decision to publish, or preparation of the manuscript."

3. Please review your reference list to ensure that it is complete and correct. If you have cited papers that have been retracted, please include the rationale for doing so in the manuscript text, or remove these references and replace them with relevant current references. Any changes to the reference list should be mentioned in the rebuttal letter that accompanies your revised manuscript. If you need to cite a retracted article, indicate the article’s retracted status in the References list and also include a citation and full reference for the retraction notice.

=> none of the cited papers has been retracted since submission. We added the 4 references that were kindly suggested by the reviewer, as well as other references to better set up the rationale of the study (in the introduction):

- Lines 45-50: References Van Limbergen Gastro 2008 and Vernier-Massouille Gastro 2008 replaced and updated by the following references: Molodecky Gastro 2012 [1], Ghione Am J Gastro 2018 [2], Sykora World J Gastro 2018 [3]

*“Inflammatory bowel disease (IBD), including Crohn’s disease and Ulcerative colitis, is a chronic relapsing disease affecting the digestive tract. The incidence and prevalence of these diseases are increasing worldwide [**1**]. A systematic worldwide review showed that the highest reported prevalence values for IBD were in Europe (UC: 505 per 100,000 persons, CD: 322 per 100,000 persons) [**1**]. An estimated ten percent of new IBD cases patients are children, with a steady increase of incidence of pediatric IBD worldwide [**2**,* *3**].”*

- Lines 50-54: Added refererences: Fumery Dig Liver Dis 2019 [5], Pigneur IBD 2010 [6], Guan J Immunol Res 2019 [7]

*“Crohn’s disease beginning in childhood has several specificities compared to adult-onset disease among which a higher incidence of complicated phenotypes and of growth failure [**4-6**]. Focusing on the mechanisms of the similarities and differences between pediatric and adult IBD could help unravel some aspects of IBD pathogenesis.”*

- Lines 58-60: Added references: Eichele World J Gastro 2017 [9], Gadaleta Curr Protoc Mouse Biol 2017 [10], Siracusa Nutrients 2020 [11]

*“Animal models of intestinal inflammation mimicking Inflammatory Bowel Disease (IBD) are widely used to better understand the cellular and molecular pathways of inflammation and fibrosis, and target these pathways to develop new drugs [**8-11**].”*

- Lines 61-63: Added reference: Neurath Int Rev Immunol 2000 [13]

*“Intra-rectal instillation of TNBS causes an IL-12-driven Th1 T-cell mediated immune response in the colonic mucosa [**13**].”*

- Lines 70-72: Added reference Rashidian Inflammopharmaco 2016 [14]

*“The model has been used to test anti-inflammatory properties of dietary compounds, and drugs on colonic inflammation through inhibition of the NF-κB pathway [**11**,* *14**].”*

- Lines 82-83: Reference Ruemmele JCC 2014 replaced by updated guideline reference: van Rheenen JCC 2020 [18]

*“The recommended treatment for inducing remission in children with IBD is exclusive enteral nutrition [18].”*

- Lines 363-364: Added references: Liu Signal Transduct Target Ther 2017 [34],

*“These inflammatory markers are part of the NF-κB signaling pathway [34].”*

- Lines 364-366 : Added reference : Mbodji J Nutr Biochem 2013 [35],

“*In our previous studies with older rats, we found that acute TNBS colitis was associated with colon NF-κB activation with upregulated downstream molecules [35].”*

- Lines 366-367 : Added reference : Di Paola Pharmacol Res 2019 [36]

*“This was in accordance with findings from pre-clinical studies using chemical-induced colitis [**11**,36].”*

Here are the point-by-point replies to the reviewer’s comments:

Reviewer #1: This study investigated the Magnetic Resonance Colonography assessment of Acute Trinitrobenzene Sulfonic Acid Colitis in pre-pubertal rats. The rational behind the experiment was clear andstraight forward. The manuscript is almost well written.
=> Thanks for your comments

While many different sources are used to set up the study in the introduction, little previous evidence is
stated. The introduction is thus short and poorly sets up the rationale for the study. More attention to how
this study fits into previous work in colitis and inflammation should be added to improve this section.
Please refer to doi: 10.3390/nu12030834 [Siracusa Nutrients 2020], 10.3109/08830180009048389 [Neurath Int Rev Immunol 2000]

=> we thank the reviewer for these references and have added them to the introduction:

- Lines 58-60: Added reference: Siracusa Nutrients 2020 [11]

*“Animal models of intestinal inflammation mimicking Inflammatory Bowel Disease (IBD) are widely used to better understand the cellular and molecular pathways of inflammation and fibrosis, and target these pathways to develop new drugs [**8-11**].”*

- Lines 61-63: Added reference: Neurath Int Rev Immunol 2000 [13]

*“Intra-rectal instillation of TNBS causes an IL-12-driven Th1 T-cell mediated immune response in the colonic mucosa [**13**].”*

There are some minor grammar issues that should be fixed in order to aid the accessibility of the results to
the reader.

=> English language was checked by a native-speaker and we made the necessary grammar corrections

The discussion does a good job at explaining the importance of the results in the context of the inflammatory
pathways involved. However, incorporation of previous results from other related studies is lacking.
Incorporating comparisons with other studies would increase the strength of the paper. Please describe
better the role of the inflammation pathway. Please refer to: doi:10.1016/j.phrs.2019.01.041 [Di Paola Pharmacol Res 2019], 10.1007/s10787-016-0263-6.

=> these references have been added to the introduction and discussion:

- Lines 366-367 : Added reference : Di Paola Pharmacol Res 2019 [36]

*“This was in accordance with findings from pre-clinical studies using chemical-induced colitis [**11**,36].”*

- Lines 70-72: Added reference Rashidian Inflammopharmaco 2016 [14]

*“The model has been used to test anti-inflammatory properties of dietary compounds, and drugs on colonic inflammation through inhibition of the NF-κB pathway [**11**,* *14**].”*

We also added a paragraph on the inflammation pathway with comparisons with studies from others and us

Lines 361-371:

“We did not observe a difference in levels of mRNA for inflammatory cytokines (IL-1β and TNFα) or NOS 2 and colon COX-2 expression between the 3 groups, although this could be due to a lack of power due to small size of study groups. These inflammatory markers are part of the NF-κB signaling pathway [34]. In our previous studies with older rats, we found that acute TNBS colitis was associated with colon NF-κB activation with upregulated downstream molecules [35]. This was in accordance with findings from pre-clinical studies using chemical-induced colitis [11, 36]. As in the present study, we previously did not observe a significant difference in colon COX-2 expression in rats with TNBS-induced colitis 7 days after the TNBS injection while colon COX-2 expression was upregulated in rats with TNBS-induced colitis after 2 days [17]. The timing of the present study (7 days after) may have contributed to this discrepancy by a partial recovery. “

We hope our revised manuscript will meet your publication criteria and thank you for your careful consideration

Best regards,

Claire Dupont-Lucas, MD PhD
